# Dynamic Changes, Spatiotemporal Differences and Factors Influencing the Urban Eco-Efficiency in the Lower Reaches of the Yellow River

**DOI:** 10.3390/ijerph17207510

**Published:** 2020-10-15

**Authors:** Yu Zhang, Wenliang Geng, Pengyan Zhang, Erling Li, Tianqi Rong, Ying Liu, Jingwen Shao, Hao Chang

**Affiliations:** 1 Research Center of Regional Development and Planning, College of Environment and Planning, Institute of Agriculture and Rural Sustainable Development, Henan University, Kaifeng 475004, China; 1426103012@vip.henu.edu.cn (Y.Z.); gengwl@henu.edu.cn (W.G.); rongtianqi@henu.edu.cn (T.R.); liuying@henu.edu.cn (Y.L.); jingwenShao@henu.edu.cn (J.S.); changhao@henu.edu.cn (H.C.); 2 Collaborative Innovation Center on Yellow River Civilization of Henan Province, Henan University, Kaifeng 475001, China

**Keywords:** eco-efficiency, Super-SBM, STIRPAT, lower reaches of Yellow River

## Abstract

The measurement of eco-efficiency is an important tool to evaluate the level of urban sustainable development. Therefore; improving urban eco-efficiency in the lower reaches of the Yellow River ensures the implementation of ecological protection and high-quality development strategies in the Yellow River Basin. In this study; the dynamic changes of urban eco-efficiency and spatiotemporal differences in the lower reaches of the Yellow River were investigated using the Super-SBM (Super-Slack measure model) model with undesirable outputs and standard deviation ellipse. The STIRPAT (Stochastic Impacts by Regression Population; Affluence and Technology) model was introduced to analyze the factors affecting the change in urban eco-efficiency. The results showed that the overall urban eco-efficiency in the lower reaches of the Yellow River has not reached the optimal level. The overall eco-efficiency in the lower reaches of the Yellow River in Shandong Province was higher than that in Henan Province but the gap is narrowing. The spatial differentiation of urban eco-efficiency in the lower reaches of the Yellow River showed the following trends: “blooming in the middle and reverse development at both ends” in the high-value area and gradual decrease in the low-value area. From 2007 to 2018; a direction was notable with respect to the development of urban eco-efficiency in the lower reaches of the Yellow River; with the centripetal force weakening. Although the mean center of urban eco-efficiency located in Shandong Province; it notably shifted to the west during the study period. In terms of driving factors; affluence and technological progress play positive roles in driving eco-efficiency; while investment intensity; industrial structure; and foreign investment intensity hindered the optimization and improvement of urban eco-efficiency in the lower reaches of the Yellow River. The results of this study show that urban eco-efficiency in the lower reaches of the Yellow River is improving; but the regional coordination is poor. The main methods promoting the sustainable development in the study area include changing the mode of extensive investments and the introduction of foreign capital; which improve the energy efficiency and promote faster and better economic development.

## 1. Introduction

As economic, political, and cultural centers, cities accommodate more than 50% of the world’s population and contribute to 80% of the gross domestic product (GDP) growth [1,2]. Urbanization has promoted economic growth and profound changes in the population structure [3]. However, unrestricted urbanization severely affects the ecological environment and leads to changes in global and regional resources, such as global warming, environmental pollution, food security, and so on [4,5]. China has experienced large-scale urbanization and industrialization since the reform and opening up (since 1976) [6]. While urbanization promotes rapid development of the social economy, it has caused serious damage to the ecological environment, which has become the key factor restricting the sustainable development of Chinese cities [6,7,8,9]. Methods to effectively maximize economic output while minimizing environmental pollution and to promote the transformation of the extensive development model of cities to an “intelligent”, “sustainable”, and “healthy“ development model have become the focus of governments and scholars worldwide [10,11,12,13].

The concept of eco-efficiency, put forward by Schaltegger and Sturm in 1990 [14], refers to a cross-efficiency that connects environmental problems with economic problems and embodies the relationship between economic development and utilization of resources and the environment [15,16]. Embedding the ecological consumption of resources into traditional socioeconomic input–output accounting provides a new cut-in point and perspective for the comprehensive study of the level and quality of urban development [17]. The World Council for Sustainable Business defines eco-efficiency as the provision of competitive goods and services to meet human needs and improve the quality of life, while gradually reducing ecological impacts and the resource intensity throughout the life cycle to meet the carrying capacity of the planet [18]. After more than 20 years of development, the evaluation of eco-efficiency has become an important tool for the comprehensive analysis of urban sustainable development and a focus of academic research pertaining to urban geography [19,20].

Scholars have conducted extensive research on eco-efficiency with respect to evaluation indicators, measurement methods, and driving factors [21]. In this study, the progress and challenges of current research as well as methods for improvement are discussed. The evaluation index of the eco-efficiency has changed from a single ratio to a comprehensive index system including resource input, energy consumption, economic output, and environmental pollution output. However, urban CO_2_ emissions are rarely included in the index system in current research, mainly because CO_2_ plays an important role in the development and sustainability of life, and thus is not regarded as a pollutant [22]. However, CO_2_ produced by fossil fuel combustion during urban development is the main carbon source leading to global warming [23] and is regarded as an undesirable output in the evaluation of eco-efficiency in this study. Based on this perspective, the measurement results better reflect the actual development processes of cities.

Efficiency is a multi-dimensional concept because the units used to measure the input and output are different [16]. Current research on urban eco-efficiency mainly focuses on life cycle analysis [24], the ecological footprint [25], frontier analysis [26], and data envelopment analysis (DEA) [27]. Although many standardized and normalized statistical methods are available, both subjective and objective methods have limitations. The DEA is widely used in the study of eco-efficiency because it is not affected by index units and weights and is easy to operate [28,29]. Research on urban eco-efficiency based on DEA is mainly divided into three aspects. First, during the processing of undesirable indicators, undesirable data generated in the process of urban production are classified into the input part to measure, which is usually based on traditional or super-efficiency DEA models [16]. Second, some scholars have used DEA model based on mixed distance function to solve the deviation of measurement results of DEA model based on radial and non-radial angle. [30,31]. Third, when faced with decisions in different directions, the traditional DEA model is not sufficiently comprehensive to measure the undesirable output (e.g., pollutants); hence, there may be a deviation in the efficiency [32]. To obtain accurate results, scholars generally process undesirable outputs using the SBM (slack measure model), which better reflects the actual situation [33,34]. However, during the calculation, multiple decision making units (DMUs) efficiency values might equal 1. In order to solve the defects, the Super-SBM model is often introduced for the calculation of eco-efficiency [35]. Because the Super-SBM model can not only measure the undesirable output but also compare the eco- efficiency of each DMU, it is the most suitable model for this study.

Third, exploring the effects of socioeconomic factors on the difference in the urban eco-efficiency is the focus of the current research. Many regression models are available to study the socioeconomic determinants of eco-efficiency, including the spatial and non-spatial regression models [31]. Because the STIRPAT (Stochastic Impacts by Regression Population, Affluence and Technology) model can accurately specify the sensitivity of environmental impacts to the driving forces, it has been widely used in the study of the effects of human activities on the ecological environment [36,37].

At present, Chinese scholars have made great progress in the study of the relationship between ecology and the economy based on the DEA model. However, research on the regional ecological environment is mainly focused on urban agglomeration [19], with a high level of urbanization, the eastern coast [21], and the Yangtze River economic belt [38] with good regional coordination. There is a relative lack of research on the eco-efficiency of the sub-region of the river basin where the level of urbanization lags and the pressure of ecological environment is high. The plain area of the lower reaches of the Yellow River is one of the most important grain-producing areas in China and has the longest farming history [39]. It is one of the typical areas of the Yellow River Basin in which the economy develops rapidly and human activities have a greater impact on the environment [28]. This is characterized by the lack of groundwater, uneven economic development of regional cities, and serious environmental pollution [40,41]. Ensuring ecological protection and high-quality economic development in the Yellow River Basin have become national strategies; further, the green transformation and development of cities in the lower reaches of the Yellow River are urgent problems to be solved. In this study, we constructed an index system for measuring urban eco-efficiency including environmental pollution data and explored the temporal and spatial evolution of urban eco-efficiency in the process of urbanization of traditional agricultural areas in China. In addition, the factors influencing the change in eco-efficiency are analyzed, which is of great significance for the sustainable economic development of cities in the lower reaches of the Yellow River.

By combing the relevant research progress of urban eco-efficiency, this study analyzes the challenges existing in the current research and puts forward corresponding solutions to make the research reflect the actual process of urban production. In this study, the lower reaches of the Yellow River, which are characterized by a contradiction between urban economic development and ecological environment protection, were selected as the study area. The estimated urban CO_2_, wastewater, sulfur dioxide, and dust emissions were included in the undesirable output—these indicators can reflect the efforts made by local governments in the improvement of water quality and atmospheric environment control. The Super-SBM model was used to measure the change characteristics of urban eco-efficiency in the lower reaches of the Yellow River during 2007–2018. Moreover, the STIRPAT model, which is relatively mature with respect to the analysis of the eco-environmental tradeoff and coordination [42,43], was introduced to determine the effect of human factors, such as population, affluence, technology, and urbanization, on urban eco-efficiency. The results support urban development and eco-environmental protection, providing references for research on the sustainable development of the region and cities with similar environmental and economic conditions worldwide.

In this study, an index system was established that can better reflect the real urban production. The most suitable measurement model of urban eco-efficiency was selected based on the characteristics of the DEA model, with the relatively mature STIRPAT model being used to measure the effects of social and economic factors of eco-efficiency. Finally, the cities in the lower reaches of the Yellow River were selected as the study objects to analyze the spatiotemporal variation and driving mechanism of urban eco-efficiency in traditional farming areas. The overall structure of this study is as follows: The data resources and method used for the estimation of eco-efficiency are described in Section 2. The changes in the time series and spatial differentiation of urban eco-efficiency in the lower reaches of the Yellow River are analyzed in Section 3, as well as the effects of socioeconomic factors, which are discussed based on the STIRPAT model of urban eco-efficiency. The novelty and shortcomings of the study as well as future research directions are discussed in Section 4. Conclusions and suggestions for the sustainable development of cities in the lower reaches of the Yellow River are provided in Section 5.

## 2. Materials and Methods

### 2.1. Study Area

**Figure 1:**
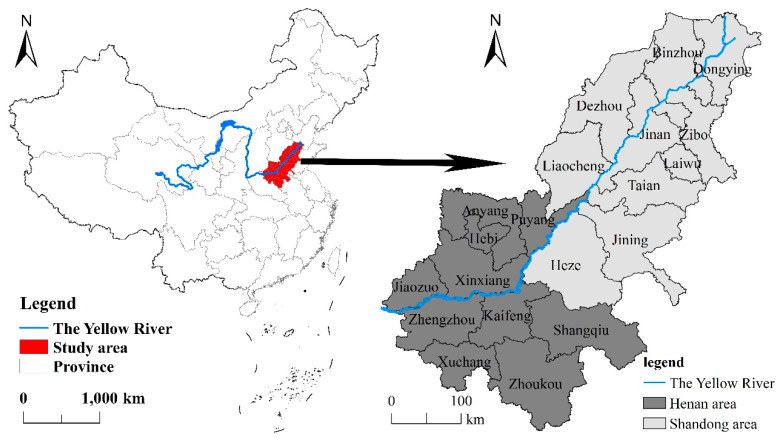
Location of the study area.

Based on the natural division, the lower reaches of the Yellow River, with a length of 785.6 km, can be divided from Taohuayu to Lijin County [43]. Because the close relationship between regional economic development in the lower reaches of the Yellow River and the integrity of administrative divisions at the prefectural level, the areas covered by the irrigation areas in the lower reaches of the Yellow River are merged into the study area [41,44] (see Figure 1). Laiwu City in Shandong Province was merged with Jinan City in 2019, forming the Laiwu District. However, because the research period of this study is 2007–2018, Laiwu City was still considered as an independent prefecture-level city. By 2018, the GDP in the study area reached 6.63 trillion yuan, accounting for more than 40% of the total GDP of the whole basin, while the average level of urbanization and urbanization level of key cities reached >57% and >70%, respectively. In 2019, the ecological protection and high-quality development of the Yellow River Basin became national strategies that provided strategic opportunities for the coordinated development of the regional economy as a unit [45]. Therefore, it is urgent to study the spatiotemporal differences of the urban eco-efficiency in traditional farming areas in the lower reaches of the Yellow River and to analyze the influencing factors in order to provide relevant policy suggestions for the study of regional urban sustainable development.

The data were obtained from the 2008–2019 “China City Statistical Yearbook”, 2008–2019 “Shandong Statistical Yearbook”, and 2008–2019 “Henan Statistical Yearbook”. In this study, we used interpolation to supplement instances of data loss. The yearbook data are obtained from China Economic and Social Big Data Research Platform [46].

### 2.2. Indicator Syste

According to previous research results; the actual situation of urban development in the lower reaches of the Yellow River; and principles of comprehensiveness, scientificity, typicality, operability, and dynamics, researchers have established an index system for the determination of the urban eco-efficiency [9,15,16,17,19]. This system is based on the principle that the DEA model’s calculation indicators are less than or equal to one-third of the number of DMU [47]. In this study, input–output evaluation indicators, including resource input, economic output, and pollution output, were established. The urban resource input includes the resources, labor, and capital and the GDP is the desirable output. Pollution data are considered as undesirable output. The specific indicators are shown in Table 1.

#### 2.2.1. Input Index

According to previous studies, the input of production factors calculated using DEA mainly includes labor, capital, and energy resources. Based on previous studies, energy is an important input resource for urban development [31]. Because the energy data of prefecture-level cities are difficult to obtain, in order to make the energy input of different prefecture-level cities comparable with each other, this study converted the consumption of natural gas, liquefied petroleum gas, and electricity consumption of different prefecture-level cities into 10,000 tons of standard coal equivalent (SCE). According to their calorific value, and then added the values up to obtain the total energy input of each prefecture-level city. Labor resources are expressed in terms of the urban employment population at the end of the year. The fixed asset investment was reduced by selecting the total fixed asset investment and using 2007 data as the constant price based on previous depreciation rates [32] and the perpetual inventory method to simulate the capital stock during the study period. The equation is as follows:

(1) $$K_{o}=\frac{I_{0}}{g+\delta }K_{t}=\left(1-\delta \right)K_{\left(t-1\right)}+I_{t}$$

where *K*_0_ represents the capital stocks of the base period, *I*_0_ denotes the gross fixed capital formation of the base period, *g* is the annual average growth rate of the regional GDP, and *δ* is the depreciation rate. We selected the depreciation rate following the research of Hall and Jones [48].

#### 2.2.2. Output Index

The output index includes desirable and undesirable output indicators. The actual GDP of each city is used as the desirable output indicator. However, due to differences in the levels of economic development of various cities, we reduced the GDP using 2007 as the base period according to the GDP index in the annual statistical yearbook to eliminate the influence of price factors and allow for comparison.

Many pollutants are generated during urbanization and industrialization. Based on the selection of traditional pollutant indicators, the city’s CO_2_ emissions were simulated in this study considering the availability and consistency of data and actual production and living conditions. For the specific method of urban carbon dioxide, see Equation (2). The result of this calculation can reflect the actual city production process better.

### 2.3. Research Method

#### 2.3.1. Urban Carbon Emission Estimation

Energy consumption is the main source of carbon emissions [23]. Because it is difficult to collect energy consumption data of prefecture-level cities in the lower reaches of the Yellow River, we followed previous studies and used the product of the output values of secondary and tertiary industries and the energy consumption per unit of GDP as urban carbon emissions [49]. The output values of secondary and tertiary industries are mainly generated by urban economic activities, and thus choosing GDP energy consumption data to calculate carbon emissions can better reflect the government’s efforts in energy saving, emission reduction, and industrial upgrading [50]. The equation is as follows:

(2) $$E_{i}=GDP_{23}\times H\times K$$

where *E_i_* represents the carbon emissions of the *i*-th city, GDP_23_ represents the GDP of the secondary industry and the tertiary industry of the *i*-th city, *H* is GDP energy consumption (standard coal/10,000 yuan), and *K* is the carbon emission coefficient of the coal consumption based on the conversion factor of standard coal into CO_2_. The value of standard coal *K* was determined to be 2.7725 t CO_2_/tce based on previous studies [51].

#### 2.3.2. Super-SBM Model Based on Undesirable Output

The DEA model as a linear programming method for measuring the technical efficiency of DMUs was first proposed by Charnes et al. in 1978, which can include multiple input–output variables [52]. In order to avoid errors when the traditional DEA model is faced with different directions of decision making, this study calculated the urban eco-efficiency of the lower reaches of the Yellow River according to the SBM model based on undesirable outputs proposed by Tone [33]. However, it was found that there were multiple DMUs whose efficiency values were equal to 1. To make the efficiency value of each DMU comparable, we introduced the Super-SBM model to measure eco-efficiency. The Super-SBM model is a new model proposed by Tone in 2002 that is based on the Super-DEA model and the SBM-DEA model, combining the advantages of the SBM model and the super-efficiency DEA model. Each DMU can be compared according to the actual situation [35,53]. First, assume that *n* decision making units (DMUs), *X* inputs, and *Y* output variables, *m*, *s*_1_, and *s*_2_ are expressed as the number of inputs, desirable output, and undesired output, respectively. Each DMU will have m types of inputs *X_ij_* (*q* = 1, 2, …, *m*), *S*_1_ desirable outputs, and *S*_2_ undesired outputs (*r* = 1, 2, …, *s*_2_). The specific formula is as follows:

(3) $$\rho^{*}=min\frac{1-\frac{1}{m}(\sum_{i=1}^{m}\frac{s_{i}^{-}}{x_{ik}})}{1+\frac{1}{S_{1}+S_{2}}\left(\sum_{r=1}^{s_{1}}\frac{s_{r}^{g}}{y_{rk}^{g}}+\sum_{r=1}^{s_{2}}\frac{s_{r}^{b}}{y_{rk}^{b}}\right)}$$

$$x_{k}-\overset{n}{\underset{j=1,\neq k}{\sum }}\lambda_{j}x_{j}+s^{-}\geq 0$$

$$-y_{k}^{g}+\overset{n}{\underset{j=1,\neq k}{\sum }}\lambda_{j}y_{j}^{g}+s^{g}\geq 0$$

$$y_{k}^{b}-\overset{n}{\underset{j=1,\neq k}{\sum }}\lambda_{j}y_{j}^{b}+s^{b}\geq 0$$

$$1-\frac{1}{S_{1}+S_{2}}\left(\overset{s_{1}}{\underset{r=1}{\sum }}\frac{s_{r}^{g}}{y_{rk}^{g}}+\overset{s_{2}}{\underset{r=1}{\sum }}\frac{s_{r}^{b}}{y_{rk}^{b}}\right)\geq \varepsilon$$

$$\lambda ,s^{-},s^{g},s^{b}\geq 0$$

where *s*^−^, *s^g^*, and *s^b^* denote the slack in the inputs, desirable outputs, and undesirable outputs, respectively. The target function value of *ρ* is the efficiency value of the DMU. When *ρ* ≥ 1, the DMU is effective.

#### 2.3.3. Standard Deviation Ellipse

The standard deviation ellipse (SDE) was proposed by Lefever in 1926 for the characterization of the spatial direction distribution of geographic elements, focusing on revealing the global characteristics of the spatial distribution of geographic elements [54,55]. The model includes 4 basic elements—azimuth, mean center (MC), standard deviation along the long axis, and standard deviation along the short axis, which indicate the main trend direction of the development of geographic elements and degree of dispersion in the main and secondary directions and represent the central position of all data [56,57,58]. The corresponding equations are as follows:

Mean center:

(4) $${\bar{X}}_{q}=\frac{\sum_{i=1}^{n}q_{i}x_{i}}{\sum_{i=1}^{n}q_{i}};{\bar{Y}}_{q}=\frac{\sum_{i=1}^{n}q_{i}y_{i}}{\sum_{i=1}^{n}q_{i}}$$

The standard deviations along the *x*- and *y*-axes can be calculated as

$$\sigma_{x}=\frac{\sqrt{\sum_{i=1}^{n}\left(q_{i}\bar{x_{i}}\cos\theta -q_{i}\bar{y_{i}}\sin\theta \right)}}{\sum_{i=1}^{n}q_{i}^{2}}$$

(5) $$\sigma_{y}=\frac{\sqrt{\sum_{i=1}^{n}\left(q_{i}\bar{x_{i}}\sin\theta -q_{i}\bar{y_{i}}\cos\theta \right)}}{\sum_{i=1}^{n}q_{i}^{2}}$$

where *x_i_* and *y_i_* are the spatial coordinates of the research object, *q_i_* is the weight, *i* is the DMU, and *x* and *y* represent the relative coordinates of each point from the center of the region.

#### 2.3.4. STIRPAT Model

The IPAT (Impact, Population, Affluence, Technology) model has been widely used to analyze the relationship between human activities and the environment since it was proposed in the 1970s (Equation (6)) [59]. Dietz et al. proposed the STIRPAT (Stochastic Impacts by Regression Population, Affluence and Technology) model based on the elasticity coefficient in 1994 [60]. The STIRPAT model (Equation (7)) more accurately measures the effects of socioeconomic factors on the environment [42,61]. The equations are as follows:

(6) I=P×A×T

(7) $$I=aP^{b}A^{c}T^{d}e$$

where *I* is the environmental impact; *P* is the population concentration; *A* is the degree of affluence; *T* is the technological progress; *a* is the constant term; *e* is the error term; and *b*, *c*, and *d* are human-driven indices of *P*, *A*, and *T*, respectively.

Based on previous studies [43], we used the STIRPAT model in this work to detect the driving factors that affect the urban eco-efficiency of the lower reaches of the Yellow River. It measures the 6 aspects of population agglomeration, wealth, technological progress, industrial structure, openness, and investment intensity. After normalization with the dependent variable, application of the natural logarithm, and regression simulation, we obtained the following equation:

(8) $$ln\;E=A_{0}+b\;ln\;P+c\;ln\;A+d\;ln\;T+g\;ln\;F\;DI+h\;ln\;I\;I+ln\;\varepsilon$$

where *E* represents the eco-efficiency of the city, *P* is the population agglomeration expressed by the level of population urbanization, *A* is the per capita GDP, and *T* is the technological progress. Based on previous studies, technological progress is mainly reflected by improvements in the energy efficiency during urban development [62]. Therefore, the reciprocal of the energy consumption per unit GDP was used in this study. The parameter FDI indicates the intensity of foreign investment, which is expressed as the ratio of the actual utilized foreign capital to the GDP. The parameter II is the investment intensity and is expressed by the ratio of fixed asset investments to the GDP. The parameters *a*, *b*, *c*, *d*, *g*, and *h* are elastic coefficients.

## 3. Results

### 3.1. Spatiotemporal Variation of the Urban Eco-Efficiency in the Lower Reaches of the Yellow River

#### 3.1.1. Temporal Changes in the Urban Eco-Efficiency in the Lower Reaches of the Yellow River

**Figure 2:**
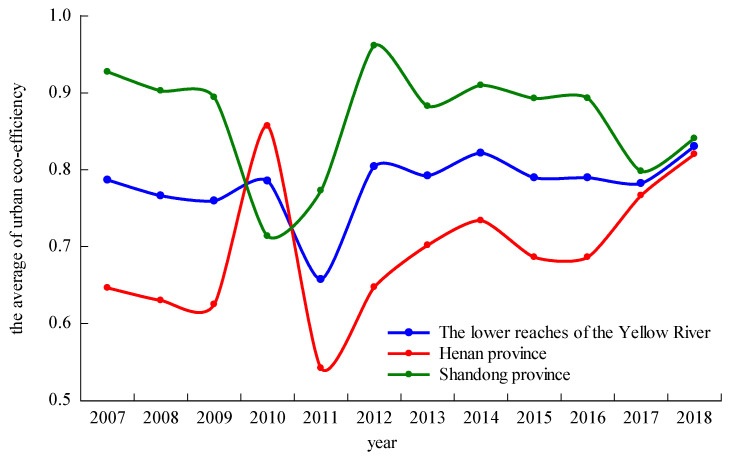
Average change of the urban eco-efficiency in the lower reaches of the Yellow River in 2007–2018.

Based on the Super-SBM model with undesirable output, we used MAXDEAultra7.2 software (Beijing Realworld Software Company Ltd., Beijing, China) to measure the urban eco-efficiency of 20 prefecture-level cities in the lower reaches of the Yellow River from 2007 to 2018 and analyzed the spatiotemporal evolution of the urban eco-efficiency in China. Figure 2 shows that the value of urban eco-efficiency in the lower reaches of the Yellow River did not reach 1, and thus the whole study area had not reached the stage of “efficiency”. The average efficiency in each year ranged from 0.6 to 0.8. The overall average eco-efficiency in the lower reaches of the Yellow River in Shandong Province was higher than that in the lower reaches of Henan Province. However, optimum efficiency had not been achieved, indicating that the focus must be placed on green development when considering the economic development of cities in the lower reaches of the Yellow River in order to improve eco-efficiency.

During the study period, the average urban eco-efficiency in the lower reaches of the Yellow River steadily increased, except for the period of 2010–2012. In 2011, the eco-efficiency of the study area was at the lowest level in the study period, and began to rebound in 2012, mainly due to the implementation of the Twelfth five-year plan for the prevention and control of air pollution in key areas in 2012, which strengthened the efforts of environmental protection and control. The urban eco-efficiency in the lower reaches of the Yellow River in Shandong Province shows an overall “decline–rise–decline” trend, while the urban eco-efficiency in the lower reaches of the Yellow River in Henan Province fluctuated in the early stage and steadily increased in the later stage. From a phased point of view, the eco-efficiencies of the cities in the lower reaches of the Yellow River showed a downward trend in the early stage of the study period (2007–2009), mainly due to the government increasing infrastructure investments and vigorously promoting the development of the economy to slow down the impact of the global economic crisis. This led to inefficient economic development and serious environmental pollution. In the middle of the research period (2009–2014), the urban eco-efficiency in the lower reaches of the Yellow River significantly fluctuated. The urban eco-efficiency in Shandong Province first declined, then rose, and then declined. The urban eco-efficiency in Henan Province first increased, then decreased, and finally steadily increased. In the later period of the research period (2014–2018), the gap in the urban eco-efficiencies between the two provinces gradually decreased. In the past two years, the level of the urban eco-efficiency in the lower reaches of the Yellow River in Henan Province has been the same as that in Shandong Province, indicating that the mode of economic development in Henan Province has changed since 2011 and the efficiency of economic development has improved on the basis of energy conservation and emission reduction, which greatly improves urban eco-efficiency.

**Figure 3:**
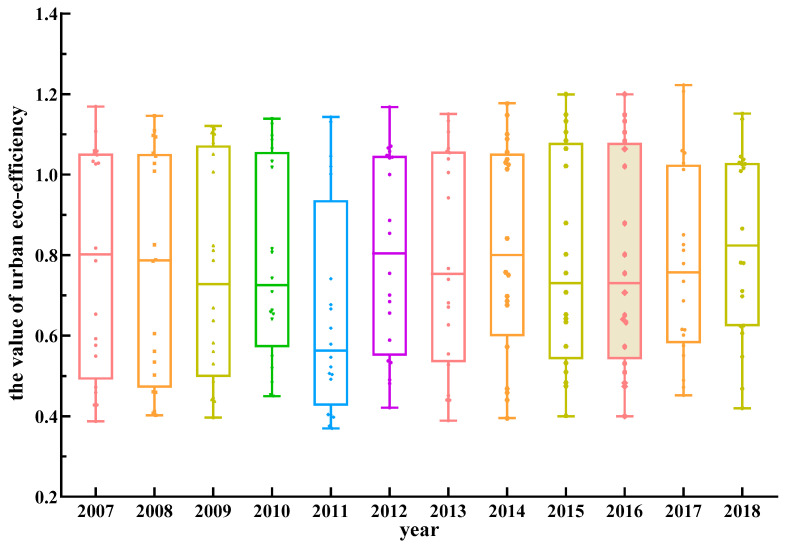
Boxplot of the urban eco-efficiency in the lower reaches of the Yellow River.

Figure 3 shows the differences in urban eco-efficiency in the lower reaches of the Yellow River from 2007 to 2018. First, the fluctuation at the upper and lower edges of the box map weakened, indicating that the high and low eco-efficiency values obtained for each year were stable. Second, except for the low efficiency value corresponding to the 75th percentile of the boxplot in 2011, the efficiency value corresponding to the 75th percentile in other years remained basically stable, and the efficiency was ~1, i.e., almost optimal. The median value decreased from 2007 to 2011 and was close to the lower edge of the box, indicating that the average level of urban eco-efficiency in the lower reaches of the Yellow River decreased during this period. During this period, urban development was mainly restricted by the traditional economic development model, the government’s economic stimulus policy, and the relatively weak environmental protection. The median distribution of the eco-efficiency fluctuated from 2011 to 2014 and gradually increased from 2015 to 2018, indicating that the average eco-efficiency improved during this period. During this period, the Chinese government promulgated a series of policies and measures to strictly protect the environment and promote the transformation of urban economic development and the upgrading of industrial structure. The gap between the upper and lower edges of the box map decreased in the past two years, indicating that the gap between the eco-efficiencies of cities has been reducing since 2017.

#### 3.1.2. Spatial Differentiation of the Urban Eco-Efficiency in the Lower Reaches of the Yellow River

**Figure 4:**
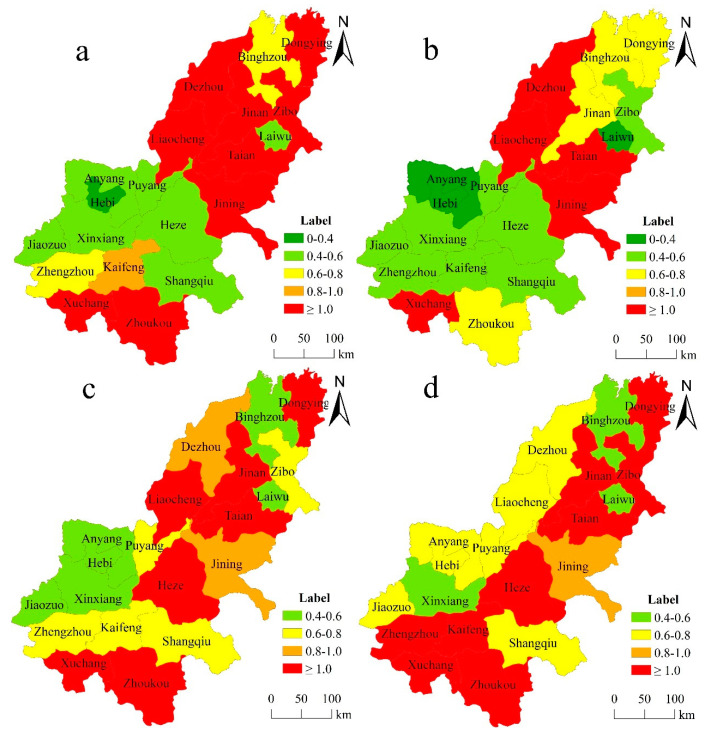
Spatial distribution pattern of the urban eco-efficiency in the lower reaches of the Yellow River ((**a**) 2007; (**b**) 2011; (**c**) 2015; (**d**) 2018).

The analysis of the temporal evolution of the urban eco-efficiency in the lower reaches of the Yellow River in Section 3.1.1 indicates a significant difference, although the difference in the eco-efficiency in the research area was alleviated later in the study period. Therefore, to explore the spatial differentiation of the urban eco-efficiency in the lower reaches of the Yellow River, we selected four time points in this study (2007, 2011, 2015, and 2018) and used the visualization tools of ArcGIS10.1 software to obtain spatial differentiation maps of the urban eco-efficiency for those years (Figure 4).

The analysis of Figure 4 shows that the level of urban eco-efficiency in the lower reaches of the Yellow River showed a deteriorating trend from 2007 to 2011, showing a trend of improvement from 2015 to 2018. The high-value area showed a “blooming in the middle and developing in the opposite direction at both ends” trend. This means the high value areas of urban eco-efficiency in Henan Province are gradually increasing, while the cities in Shandong Province with high eco-efficiency show a downward trend. In the border area of the two provinces, the urban eco-efficiency also showed improvement. Meanwhile, the urban eco-efficiency in the low-value area gradually decreased. In 2007, the high-value area in the lower reaches of the Yellow River was in Shandong Province, and only Xuchang and Zhoukou in Henan Province exhibited efficiency values greater than 1. The junction of Shandong and Henan provinces has become a low-value agglomeration area with respect to eco-efficiency. Hebi was the city with the lowest eco-efficiency in the study area (Figure 4a). In 2011, areas with low eco-efficiency expanded and the efficiency values of Zhengzhou, Zhoukou, Jinan, Dongying, and Zibo significantly decreased. The eco-efficiency values of Anyang and Laiwu declined below 0.4 (Figure 4b). In 2015, the high-value area rebounded and the area with an eco-efficiency below 0.4 gradually disappeared. The high-value area in Henan Province returned to the state of 2007. Dezhou City in the Shandong Province did not reach an efficient level, but the urban eco-efficiency of Heze City reached the optimum level (Figure 4c). In 2018, the range of areas with low eco-efficiency decreased, areas with high eco-efficiency in Henan Province expanded, and Zibo City in the Shandong Province reached an efficient level, while the eco-efficiency of Liaocheng City decreased (Figure 4d).

**Figure 5:**
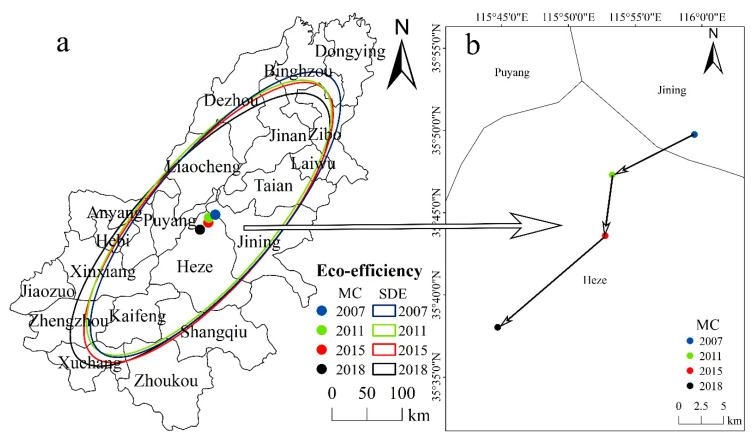
Standard deviation ellipse of the urban eco-efficiency and mean center transfer in the lower reaches of the Yellow River. (**a**) Distribution of the standard deviation ellipse of the urban eco-efficiency in the lower reaches of the Yellow River. (**b**) Mean center transfer curve of the urban eco-efficiency in the lower reaches of the Yellow River.

The analysis of the temporal–spatial characteristics of urban eco-efficiency is of great significance to the study of the stage and spatial differentiation characteristics of urban eco-efficiency in the lower reaches of the Yellow River. Therefore, to analyze the spatial evolution of the urban eco-efficiency in the lower reaches of the Yellow River during the research period, we used the SDE model and MC in this study. On the basis of the urban eco-efficiency in the study area in 2007, 2011, 2015, and 2018 and through using ArcGIS10.2 software, we obtained the spatial pattern of the urban eco-efficiency in the lower reaches of the Yellow River (Figure 5a), the MC migration curve of the eco-efficiency (Figure 5b), and quasi-elliptical parameters and changes in the eco-efficiency MC (Table 2).

The results of Table 2 show that the spatial distribution of urban eco-efficiency in the lower reaches of the Yellow River from 2007 to 2018 had a “southwest–northeast” direction, which is consistent with the overall trend in the study area and the position and trajectory of the Yellow River itself. The main axis of the SDE of urban eco-efficiency and the MC of eco-efficiency moved westward, which indicated that the cities located in the lower reaches of the Yellow River in Henan Province are characterized by better development of urban eco-efficiency and contribute more to the development of regional eco-efficiency. Based on the distribution pattern of the SDE (Figure 5a), the standard deviation of the long axis was always larger than that of the minor axis, indicating a notable development of the urban eco-efficiency in the lower reaches of the Yellow River. Further, on the basis of the results of the calculation of the SDE parameter (Table 2), the standard deviation along the *x*-axis increased from 88.66 to 89.22 km from 2007 to 2011 and the standard deviation along the *y*-axis decreased from 255.24 to 246.574 km, indicating that the development of the urban eco-efficiency was weakened and centrifugation had a notable effect during this period. The standard deviations of the *x*- and *y*-axes increased in 2015 and 2018. From 2011 to 2017, the standard deviations increased by 1.39 and 5.94 km along the *x*- and *y*-axes, respectively, indicating that the development of urban eco-efficiency in the lower reaches of the Yellow River had strengthened since 2011, while the centrifugation effect was weakened.

Based on the change in the MC transfer curve of the urban eco-efficiency (Figure 5b), we found that the MC of urban eco-efficiency was in Shandong Province but notably shifted to the west. The results show that the sustainable development of cities in Shandong Province reflected the main development of urban eco-efficiency in the study region. However, the transformation and development of cities in the lower reaches of the Yellow River in Henan Province showed good trends, contributing to the spatial evolution of urban eco-efficiency in the study region.

### 3.2. Driving Mechanism of the Urban Eco-Efficiency in the Lower Reaches of the Yellow River

#### 3.2.1. Selection of Impact Indicators

Factors influencing the urban eco-efficiency have been studied extensively. Based on the STIRPAT model, the effect of human activities on the natural environment can be analyzed by considering three aspects: population, affluence, and technological progress. The STIRPAT model was used in this study to analyze the effects of the affluence (A), population accumulation (P), industrial structure (IS), intensity of foreign investment (FDI), technology progress (T), and investment intensity (II) on urban eco-efficiency. Prior to the analysis in this study, we standardized the selected independent and dependent variables and applied a natural logarithm. The results of the analysis are shown in Table 3.

#### 3.2.2. Analysis of the Driving Mechanism

To solve the problem of inaccurate regression results caused by collinearity between data, we analyzed the collinearity between independent and dependent variables in this study. The test results VIF (variance inflation factor) were less than 7.5 empirical values [63], indicating that there was no multicollinearity problem among the variables. Based on the analysis results in Table 3, except for the urbanization level index (P), all indicators passed the significance test. The per capita GDP (A), investment intensity (II), intensity of foreign investment (FDI), and technological progress (T) were significant at the 1% significance level and the proportion of industrial added value to the GDP (IS) passed the 10% significance level test. The coefficients of the affluence and technological progress were 0.2202 and 0.5217, respectively, indicating that every 1% increase in the per capita GDP and 1% reduction in the energy consumption per unit GDP will lead to a 22.02% and 52.17% increase in urban eco-efficiency, respectively. The investment intensity, foreign investment intensity, and industrial structure coefficient were −0.2005, −0.102, and −0.159, respectively, indicating that a 1% increase in the proportion of fixed asset investment in the GDP, proportion of foreign capital actually utilized in the GDP, and proportion of industrial added value in the GDP will lead to a 20.05%, 10.2%, and 15.9% decrease in urban eco-efficiency, respectively.

On the basis of the analysis in Table 3, it is clear that the per capita GDP and technological progress effectively improved the urban eco-efficiency in the lower reaches of the Yellow River. Note that the positive effect of technological progress was more significant because technological progress is mainly reflected in the improvement of the energy use efficiency [11]. Energy is not only the basic guarantee of the life of urban residents and production but also an important source of environmental pollution. The implementation of the national strategy of eco-environmental protection and high-quality development in the Yellow River Basin will greatly improve urban energy use efficiency, change energy use structure, and reduce energy consumption per unit GDP, which can effectively improve urban eco-efficiency and promote urban sustainable development. The investment intensity, foreign investment intensity, and index of the industrial structure negatively affect urban eco-efficiency. The main reason for these negative effects is that the industrial structure in the current process of urban industrialization in the lower reaches of the Yellow River is unreasonable and there are many industries with high-energy consumption, low efficiency, and high emissions, which lowers the urban eco-efficiency. The cities in the lower reaches of the Yellow River are still in the stage of high-speed urbanization and industrialization and the extensive investment-driven model is one of the reasons for the low urban eco-efficiency. Furthermore, the process of introducing foreign investments to promote urban economic development in the lower reaches of the Yellow River includes a type of industrial transfer characterized by “The Hypothesis of Pollution Haven”, i.e., the transfer of industries from developed areas to undeveloped areas is usually dominated by high energy-consuming and high-pollution industries. These factors cause the decrease in urban eco-efficiency in the lower reaches of the Yellow River.

## 4. Discussion

Unrestricted urban development causes serious resource and environmental problems; therefore, the promotion of urban sustainable development is an important goal [64]. According to the basic connotation of eco-efficiency, research on the evolution and differences of urban eco-efficiency in the lower reaches of the Yellow River is of great significance in terms of analyzing the stages of urban development and determining future directions of urban development. First, the undesirable output in the process of urban economic production was improved in this study and the urban CO_2_ emissions were estimated based on the ratio of energy consumption per unit GDP to the second and third output value, yielding an urban efficiency that better reflects the urban economic production. Second, the Super-SBM model, which can be used to directly measure the undesirable output, was used. It combines the advantages of the SBM and Super-DEA models and eliminates the limitations of traditional index analysis. Each decision making unit was analyzed to determine the differences in the eco-efficiency among regional cities. Finally, the lower reaches of the Yellow River were selected as the study area. This area is characterized by a contradiction between urban economic development and ecological environment protection. By measuring the regional eco-efficiency and analyzing the effects of socioeconomic factors on the eco-efficiency, we were able to investigate the reasons for the differences in urban eco-efficiency in this study. Subsequently, a scientific basis was provided for the formulation of policies to promote sustainable urbanization in areas characterized by a contradiction between urban economic development and ecological environment protection.

The value of urban eco-efficiency in the lower reaches of the Yellow River did not reach 1, and thus the whole study area did not reach the stage of “efficiency”. Urban eco-efficiency in Shandong Province was generally found to be better than that in Henan Province, which is consistent with the results of previous research [65,66]. This shows that the urban development is still in the stage of high resource consumption and high environmental impact in areas with the most active urban economic development in the Yellow River Basin and is facing environmental pressure. The urban eco-efficiency is closely related to urban economic development, which can be confirmed by the analysis of driving forces, moreover validating previous research [67,68].

The development of urban eco-efficiency in the lower reaches of the Yellow River can be divided into stages, which are mainly affected by the government’s economic policies [69,70]. In the early period (from 2007 to 2011), the economic development model of the cities in the lower reaches of the Yellow River was a single traditional GDP-oriented development model and the input–output conversion rate was low. To alleviate economic risks caused by the global financial crisis, the local government implemented a series of strategies to stimulate economic development. Although these measures successfully alleviated the risk of financial crisis, environmental management during this period was relatively inefficient, which caused serious damage to the ecological environment, serious environmental problems, and the decline of urban eco-efficiency. This also reflected the overall development of urban eco-efficiency in China during this period [71]. After 2012, the government strengthened the environmental protection and governance when facing overcapacity, inefficient use of resources, and serious environmental problems. With the implementation of new urbanization and ecological civilization construction strategies, the urban development of the study area entered a new stage.

There was a significant spatial difference in the urban eco-efficiency in the study area. During the study period, the number of cities in Henan Province that reached an optimum urban eco-efficiency increased, while the number of cities with an urban eco-efficiency above 1 in Shandong Province decreased, indicating a trend of “blossom in the middle and reverse development at both ends” in the high-value area and a gradual decrease in the low-value area. Urban eco-efficiency in Henan Province is developing, leading to a westward shift of the MC of the urban eco-efficiency in the research region, which is consistent with the latest research results of Cui [72]. However, the eco-efficiency of many cities in the study area is still not optimal. Because the development of cities in the lower reaches of the Yellow River is seriously affected by administrative regionalization and the distance from the core cities, a positive spillover effect of cities with a higher urban eco-efficiency cannot be observed in the border area of Henan and Shandong.

The STIRPAT model more accurately specifies the sensitivity of environmental impacts to the forces that drive them. Therefore, the STIRPAT model was used in this study to analyze the effects of population, affluence, technological progress, industrial structure, investment intensity, and foreign capital utilization intensity on urban eco-efficiency. The results show that all influencing factors passed the significance test, except for the population elements represented by the population urbanization index. Affluence and technological progress positively affect urban eco-efficiency, but the industrial structure and investment intensity hinders the improvement of the urban eco-efficiency, which is consistent with previous research results [33,68,73,74]. In contrast to relatively developed urban areas [75], the intensity of foreign investment hinders the optimization of urban eco-efficiency in the lower reaches of the Yellow River, mainly because the industrial transfer introduced by the cities in the lower reaches of the Yellow River is related to industries with high consumption and a low production capacity.

However, this study has its limitations. Because of the small sample size, some mature spatial analysis models cannot be applied, such as exploratory spatial data analysis (ESDA), GWR (geographical weighted regression), and some spatial econometric models. In the future, we are going to take the county-level cities in the lower reaches of the Yellow River as the research region to expand the sample size and explore the spatial spillover of urban eco-efficiency and the spatial heterogeneity of driving factors in the lower reaches of the Yellow River.

## 5. Conclusions

The plain area of the lower reaches of the Yellow River in China is a traditional agricultural area. From 2007 to 2018, the area of cultivated land in the study area remained stable, accounting for about 5.6% of the total area, with grain output increasing by nearly 20% and per capita grain being 0.62 kg, ensuring food security in the lower reaches of the Yellow River. Promoting high-quality urban development under the premise of ensuring food security will remain as the theme of urban development in the future. However, the level of urban development lags compared with the developed areas in the east. Moreover, the mode of economic development that has not yet been fully transformed and the backward industrial structure have led to a prominent contradiction between urban economic development and ecological environment protection in this area, while the related research on the eco-efficiency of the Yellow River Basin is still very weak. This study enriches research on urban sustainable development in traditional agricultural areas. It is of great significance to study the dynamic changes, differences, and factors influencing the urban eco-efficiency in traditional farming areas. Based on data obtained from 2007 to 2018, we extended the study of urban eco-efficiency in this work to traditional farming areas, calculating the urban eco-efficiency of the lower reaches of the Yellow River by using the Super-SBM model. By investigating the dynamic changes and spatial differences, we determined the effects of relevant socioeconomic factors on urban eco-efficiency in this study. The results show that the overall eco-efficiency of the study area has not reached the optimal level. Although the overall urban eco-efficiency of Shandong Province is better than that of Henan Province, the urban eco-efficiency of Henan Province has been improving and the gap between the two provinces continues to decrease, leading to a continuous westward shift of the center of eco-efficiency in the study area. In addition, urban economic development and technological progress promote urban eco-efficiency, while high consumption, low-output industrial structures, extensive investment, and low environmental benefits of the foreign investment model hinder the optimization of urban eco-efficiency in the study area.

Based on the results of this study, we provide some policy suggestions for the cities in the lower reaches of the Yellow River:

(1) Changing the mode of economic development and promoting high-quality development of urban economy is the theme of urban sustainable development in the lower reaches of the Yellow River. At the same time, we should take the improvement of urban eco-efficiency as an important part of government work, and change the traditional single GDP-oriented development model in order to release the space and potential of urban green development and promote urban sustainable development.
(2) Although the gap of urban eco-efficiency between Shandong Province and Henan Province in the lower reaches of the Yellow River has been alleviated in recent years, we should still pay attention to the coordinated development between regions. The areas with high efficiency should give full play to the positive spillover effect and encourage the flow of advanced technology and industry to promote the coordinated development of cities in the river basin.
(3) The local government of the study area should pay attention to the optimization and upgrading of industrial structure and the adjustment of energy utilization structure. By reducing the proportion of fossil fuels and energy consumption per unit of GDP, the government should strive to ensure high-quality urban economic development, let people enjoy more benefits brought about by high-quality development, and improve people’s living standards.
(4) At present, urban eco-efficiency in the lower reaches of the Yellow River has been restricted by an extensive investment-driven model, and high pollution an inefficient industrial structure, and a foreign investment model with high pollution risk. Therefore, the local government should strictly abide by the environmental protection policies and regulations issued by the central government; issue and implement the “three lines and one order” as soon as possible; and build regional cooperation mechanisms for resource conservation and eco-environmental protection, as well as formulate energy conservation and emission reduction policies from a regional and urban perspective. We should give full play to the government’s “visible hand” and improve the access standards of enterprises, as well as promote high-quality development of the regional economy and society with high-level protection of the ecological environment. At the same time, we should give full play to the leading role of Zhengzhou and Jinan as national central cities, speed up the construction of the Zhongyuan urban agglomeration and the Shandong Peninsula urban agglomeration, optimize the industrial layout, and form a joint force for development. Finally, cities should avoid inefficient industries and pay attention to the introduction of high-tech industries; seize the strategic opportunities of 5G, Internet +, and block chain; increase support for local emerging industries; adjust measures to local conditions; and develop local characteristic industries so as to optimize and adjust traditional leading industries and promote urban transformation and development.

## Figures and Tables

**Table 1:** Input and output indicator statistics, 2007–2018. *n* = 240.

Category	Variable	Units	Mean	Standard Deviation (SD)	Minimum	Maximum
Input	Labor force	10^4^ people	57.109	36.032	13.27	207.555
	Capital	10^4^ yuan	6608.376	6480.381	165.322	42,605.902
	Energy resource	10^8^ tons of standard coal equivalent	184.698	192.969	12.268	1581.735
Desirable output	Gross Domestic Product	10^9^ yuan	1156.283	676.978	266	3868.797
Undesirable output	Total wastewater emission	10^4^ tons	10,546.359	7273.71	766	66,452
	Industrial SO_2_ emission	ton	61,986.833	42,586.623	917	219,273
	Industrial soot emission	ton	29,395.089	30,861.296	775	236,000
	CO_2_ emission	ton	2920.640	1869.933	678.514	9998.306

**Table 2:** Standard deviation ellipse of the urban eco-efficiency in the lower reaches of the Yellow River for the period of 2007–2018.

Year	The Standard Deviation along the Short Axis (km)	The Standard Deviation along the Long Axis (km)	Azimuth
2007	88.658	255.235	40.27
2011	89.222	246.574	40.723
2015	89.671	251.485	40.738
2018	90.612	252.518	43.232

**Table 3:** Driving factors of urban eco-efficiency.

Variable	Coefficient	Standard Error	*t*-Statistic	Probability	VIF
Constant	−0.143	0.070	−2.047	0.042 **	
Affluence	0.220	0.033	6.584	0.000 ***	1.423
Investment intensity	−0.201	0.052	−3.839	0.000 ***	1.162
Intensity of foreign investment	−0.102	0.017	−5.863	0.000 ***	1.182
Industrial structure	−0.159	0.096	−1.651	0.100 *	1.941
Technology progress	0.522	0.046	11.391	0.000 ***	1.939
Population accumulation	−0.053	0.040	−1.328	0.186	1.214

Note (1) *R*^2^ = 0.520926, *F*-statistic = 42.22583, Probability (*F*-statistic) = 0.0000; *R*^2^, *F*-statistic and Probability (*F*-statistic) are all indexes to evaluate the simulation effect of STIRPAT model, The *R*^2^ represents the coefficient of determination of the regression model, and refers to the degree of fitting of the model. The higher the value, the better the simulation effect of the model. (2) The symbols ***, **, and * denote the significance at 1%, 5%, and 10%, respectively; (3) VIF is an acronym for variance inflation factor.
